# A diachronic perspective on citation latency in Wikipedia articles on CRISPR/Cas-9: an exploratory case study

**DOI:** 10.1007/s11192-023-04703-8

**Published:** 2023-05-14

**Authors:** Marion Schmidt, Wolfgang Kircheis, Arno Simons, Martin Potthast, Benno Stein

**Affiliations:** 1 German Center for Higher Education Research and Science Studies (DZHW), Berlin, Germany; 2 Leipzig University and Center for Scalable Data Analytics and Artificial Intelligence (ScaDS.AI), Leipzig, Germany; 3 Technische Universität Berlin, German Center for Higher Education Research and Science Studies (DZHW), Berlin, Germany; 4 Bauhaus-Universität Weimar, Weimar, Germany

**Keywords:** Wikipedia, Publication matching, CRISPR, Timeliness, Relevance, Bibliometrics

## Abstract

This paper analyzes Wikipedia’s representation of the Nobel Prize winning CRISPR/Cas9 technology, a method for gene editing. We propose and evaluate different heuristics to match publications from several publication corpora against Wikipedia’s central article on CRISPR and against the complete Wikipedia revision history in order to retrieve further Wikipedia articles relevant to the topic and to analyze Wikipedia’s referencing patterns. We explore to what extent the selection of referenced literature of Wikipedia’s central article on CRISPR adheres to scientific standards and inner-scientific perspectives by assessing its overlap with (1) the Web of Science (WoS) database, (2) a WoS-based field-delineated corpus, (3) highly-cited publications within this corpus, and (4) publications referenced by field-specific reviews. We develop a diachronic perspective on citation latency and compare the delays with which publications are cited in relevant Wikipedia articles to the citation dynamics of these publications over time. Our results confirm that a combination of verbatim searches by title, DOI, and PMID is sufficient and cannot be improved significantly by more elaborate search heuristics. We show that Wikipedia references a substantial amount of publications that are recognized by experts and highly cited, but that Wikipedia also cites less visible literature, and, to a certain degree, even not strictly scientific literature. Delays in occurrence on Wikipedia compared to the publication years show (most pronounced in case of the central CRISPR article) a dependence on the dynamics of both the field and the editor’s reaction to it in terms of activity.

## Introduction

For some time now, the bibliometric community has been increasingly engaging with new data sources, often involving new databases or, as in the case of Altmetrics, social media such as Twitter. Wikipedia, however, receives less attention as a format of science communication and knowledge transfer. Yet Wikipedia is one of the most widely used internet sources and strongly connected to the scientific publication system: According to Wikipedia's own guidelines, all science-based claims must be supported by scientific references, preferably secondary over primary sources.^1^ First evidence, based on text similarity, points to the possibility that Wikipedia might in turn also have an impact on the production of new scientific publications (Thompson & Hanley, 2018). Doubts about the validity remain (Jelmeniak & Aibar, 2016), especially given the fact that editors are not expected to necessarily have academic credentials. Nevertheless, Benjakob and Aviram (2018) argue that the collective editorial process could be similar to academic review. Based on these guiding principles and further hints, Wikipedia could be very well compared to a Living Review (Elliot et al., 2014).

This raises the question to what extent Wikipedia, with its specific governance structures, produces output that in fact resembles familiar formats of scientific publication—and where it differs significantly. Our aim is to explore Wikipedia as a format of science communication, focusing on the analysis of the referenced literature in Wikipedia articles.^2^ Several quality dimensions, such as comprehensiveness, accuracy, and currency, have been proposed as evaluation measures. The quality of Wikipedia articles has been initially approached rather qualitatively in comparison to other encyclopedias. However, for an assessment of the reference structures we need more specific analyses both on conceptual and technical levels.

### Case study

Our first methodical decision is to base our research setting on a case study, which facilitates an explorative approach in terms of methods as well as content, as the distinct subject matter makes it easier to look at details where appropriate. For this purpose we chose the scientific innovation of CRISPR/Cas9 (clustered regularly interspaced short palindromic repeats/CRISPR associated protein 9). Since innovation processes are characterized by the fact that they transfer scientific knowledge into application contexts and thus connect further areas of society and public perception, Wikipedia is an interesting format in this regard. Innovation processes are also often characterized by a temporary acceleration of dynamics, which makes an interesting case for Wikipedia’s promise of timeliness and constant updating. The chosen innovation, a new gene editing technology based upon an adaptive immune system of e.g. bacteria, for which Jennifer Doudna and Emmanuelle Charpentier received the Nobel Prize for Chemistry 2020, began more than twenty years ago and has left a long paper trail of scientific publications. It provides the necessary material as well as temporal scope for our endeavor.

### Relevance

Quality dimensions applicable to the coverage (or selection) of referenced literature are comprehensiveness and relevance. Both dimensions are currently not straightforward to operationalize. What would comprehensiveness mean given the size and internal differentiation of the knowledge body on CRISPR? Relevance reflects the publications’ significance and importance for audiences, potentially dependent on further contextual factors. Coverage policies of databases as well as secondary publication formats can be understood as relevance decisions. This quality dimension is often exchanged with impact, resulting in models with high impact journals as predictor variables for Wikipedia references (Teplitskiy et al., 2015). While this may be a pragmatic solution for large-scale studies, the proxy character of this metric is particularly indirect. In order to assess this dimension and thus the resemblance of Wikipedia’s inclusion practices to that of scientific sources and resources in absence of a gold standard, we use a comparative approach: As part of our methodical settings, we relate the references of Wikipedia’s central article on CRISPR to several layered reference corpora. On the broadest level, we investigate to what extent the literature selection of Wikipedia’s central article on CRISPR^3^ is covered by the Web of Science (WoS) and corresponds to a basic field delineation of publications on CRISPR in the WoS, i.e., we use the inclusion in WoS as one possible standard delineation of scientific literature as such and assess the proximity to the core subject. As it is a specific feature of Wikipedia that its audience extends to the general public, this could be reflected in how often scientific versus other publications are referenced. As additional perspectives we assess to which extent references of the central CRISPR article concur with a basic bibliometric metric on the one hand and with the references of a selection of review papers on the other, this way complementing a metric perspective with a more semantic and diachronic perspective.

### Timeliness/Latency

As a central dimension of our analysis, we explore latency based on an expanded set of CRISPR-related Wikipedia articles. In particular, we analyze how quickly references to publications occur for the first time on one of these sites, and if they are picked up at the same time or with additional delays between Wikipedia articles. Additionally, the date of first occurrence is also related to the citation distributions of these publications in WoS. We assess the recognition delay by Wikipedia editors in relation to the delay of the citing community. Special focus is on the dates and occurrence patterns of seminal publications that we have found as being crucial for the course of the innovation, so that the quality dimension is also included in this part of our analysis. This heuristic delineation is based on both the central Wikipedia article’s history section and the review papers, thereby avoiding the prioritization of a resource here.

### Technical

On a technical level, we developed and validated a number of matching heuristics in order to find references and, in particular, to reliably identify their first occurrence within thousands of article revisions. We decided to match external data against Wikipedia instead of extracting Wikipedia’s references and matching them against external resources. The motivation behind this decision is to (1) to prohibit our analysis from being affected by assumptions about the coherence of reference formatting styles on Wikipedia, and (2) to prohibit the exclusion of references cited solely in the text. Finally, the case study format, with still manageable publication and data numbers, allows us to delve deeper into certain phenomena and issues related to data quality and referencing patterns on Wikipedia. We expect that the findings obtained will also be instructive for macro studies.

## Related research

Related research on science communication on Wikipedia often focuses on quality dimensions, such as comprehensiveness, currency, readability, or accuracy (Mesgari et al., 2015). The latter was addressed early on in various case studies. For example, Giles (2005) compared selected Wikipedia articles with the Encyclopedia Britannica and concluded that Wikipedia comes close to the expert-written traditional encyclopedia in terms of accuracy. This finding was confirmed by Casebourne et al. (2012). Reavley et al. (2012) offer similar insights from a field-specific study using the Encyclopedia Britannica, textbooks, as well as other sources for comparison: Content was rated according to accuracy, up-to-dateness, breadth of coverage, referencing, and readability, with Wikipedia being generally most highly rated except in terms of readability. In another case study on climate science, Estevez and Cukierman, (2012) found that Wikipedia tends to reflect hegemonic scientific consensus. Garcia del Valle et al. (2018) evaluate Wikipedia as a data source for text mining, with a positive outcome: They show that the similarity between diseases calculated with data from Wikipedia has a precision similar to what was obtained using PubMed. Joorabchi et al. (2020) investigate whether medical Wikipedia articles link to the Cochrane database, concluding that more important articles are more likely to cite a Cochrane review. They also propose a linking tool to Cochrane to assist Wikipedia volunteers. Arroyo-Machado et al. (2020) compare at large scale the scientific literature referenced in Wikipedia to Scopus and conclude that certain fields have a stronger relative performance in Wikipedia than in Scopus. Colavizza (2020) determines that Wikipedia represents the topical structure of COVID literature in a proportionate manner, based on several clustering and topic modeling approaches. However, Grabowski and Klein (2023), in a recent paper that has also been discussed in the general media, claim a distortion of historical facts by a group of biased editors. Several approaches focus on predictors for referencing patterns or correlations between citations from Wikipedia and established science metrics: Teplitskiy et al. (2015, 2017) name the impact factor and open access as the most significant predictors for citations to journals on Wikipedia. Colavizza (2020) identifies certain publication venues, primarily popular field-specific journals (*Lancet*, *BMJ*) as well as mega journals (*Science*, *Nature*) in his recent study on COVID-19, besides citation counts and Altmetrics. Jemielniak et al. (2019) determine that the Cochrane Database, followed by top journals like NEJM, *Lancet* and *Nature*, are the most referenced in Wikipedia medical articles. In a study on Psychology journals, Banasik-Jemielniak et al. (2021) observe a significant positive association between the *Scimago Journal Ranking* and citations to journals in Wikipedia. Besides, there is evidence that publication types other than journal articles are also referenced, as Benjakob and Aviram (2018) show in a case study on a small research strand. However, according to Singh et al. (2021), who take a macro perspective, unspecified web content is cited more often than journal articles or books. Arroyo-Machado et al. (2020) observe at the journal level a similar distribution to Scopus. Shuai et al. (2013) observe that papers, authors, and topics that are mentioned in Wikipedia have higher academic impact than those that are not mentioned. From the opposite perspective (but not restricted to scientific contents), Redi et al. (2019) identify in a mixed-methods approach sentences in Wikipedia that need citations. Not surprisingly, they conclude that these are historical facts, statistics or data about a subject, or direct or reported quotations. However, Wikipedia citations are considered as being too rare to be used for an alternative impact indicator (Kousha & Thelwall, 2017).

The reports on citation latency present a mixed picture. For the top biomedical journal papers in the study by Jemielniak et al. (2019) on medical Wikipedia articles, it took only about three months to be referenced in Wikipedia. However, Banasik-Jemielniak et al. (2021) observe an average of 6,176 days for a psychology publication to be referenced in a Wikipedia article, but this long time is partly explained by the fact that works are included that have been published before Wikipedia started. The authors explain the substantial variance across journals with the assumption that research topics which are more interesting for lay readers are much quicker transferred to Wikipedia. From the fine-grained perspective of a case study on a very small research field (the circadian clock), Benjakob and Aviram (2018) report a medium citation latency of five years. They pay specific attention to the partly belated recognition of some key findings, which they see largely in accordance with the dynamics of the respective field. According to Colavizza (2020) in his large-scaled study on COVID, the newly inserted references indeed mostly refer to the surge of COVID-related research in 2020, but he also observes that gaps in older literature are filled at the same time. In an early study dedicated to the development of a protocol for characterizing the referencing dynamics Chen and Roth (2012) suggest a non-trivial interaction between article length, age (in terms of the number of revisions), and reference density. A probable factor may be that the substantiation of articles reinforces itself, e.g. by attracting qualified and committed editors. In order to analyze whether varying latency is connected to the specific properties and dynamics of research strands, we consider an explorative approach on the domain level as very appropriate.

Other research approaches address conditions under which references are clicked (Piccardi et al., 2020), as well as feedback loops from Wikipedia back into scientific text production (Thompson & Hanley, 2018). In addition, the related datasource Wikidata has been proposed and researched as a resource for scientific information tracing (Turki et al., 2022). References in Wikipedia are often extracted or matched via identifiers such as DOI or PMID (Colavizza, 2020). This also applies to the datasets published by Redi and Taraborelli (2018) and Singh et al. (2021), the latter dataset also being used by Yang and Colavizza (2021). The extraction and matching details are not always elaborated (e.g., Arroyo-Machado et al., 2020; Priem et al., 2012). By contrast, Zagorova et al. (2020) compare in a large-scale analysis a text-based approach based on complete references with the sole usage of identifiers.

However, not only is their setting restricted to a single textual approach, but it is also limited to references categorized by Wikipedia as such, that is, excluding informal references in text segments. Pooladian and Borrego (2017) retrieve Wikipedia references in the library and information science domain manually, and note a lack of standardization and incompleteness of Wikipedia references.

Therefore and in extension of an earlier work (Schmidt et al., 2021), in order to assess Wikipedia’s referencing accuracy in more detail and to be able to gauge the required level of error tolerance in matching publications to Wikipedia, we develop and manually evaluate different exact and fuzzy matching heuristics based on identifiers, titles, and author data. We match against Wikipedia references as well as against text segments, and we evaluate the matching results with respect to the number of matches, precision values, and delays. Apart from this methodical dimension, we expand the state of research with a better understanding of how referencing patterns in Wikipedia evolve over time, and how they relate to internal perspectives on a field, based on a differentiated bibliometric perspective on the CRISPR/Cas9 case.

## Materials and methodology

**Fig. 1:**
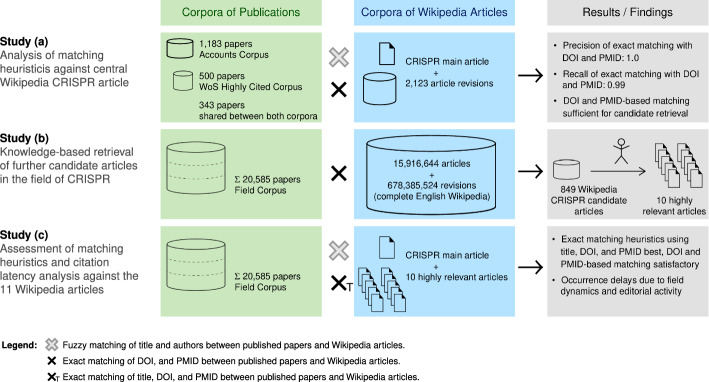
Overview of the research setting. From the pilot study (**a**), we learn the effectiveness of our algorithms for fuzzy and verbatim matching. With study (**b**), we identify the 10 most relevant CRISPR articles in Wikipedia besides the central article. Study (**c**) then forms the actual main analysis, where we quantify when and how the papers of the field corpus are considered in Wikipedia

The initial analysis shows that the formatting of references varies significantly between articles (and even between revisions of the same article) and that publications can also be cited in the text instead of in the article's reference list. While recent revisions of articles usually apply Vancouver style for the formatting of references, Wikipedia does not have a single house style but expects the editors to use a consistent style within articles, which results in citation styles changing over the course of an article's history. To avoid limiting ourselves to the extraction of references, we focus on the development of heuristics for matching external publications against Wikipedia. In a first step we created two small corpora—highly cited publications and references of a selection of review papers—to be matched with the central Wikipedia article on CRISPR in order to evaluate different matching methods (Study (a)). Having found that DOI and PMID are quite effective, we collected additional Wikipedia candidate articles by comparing the DOIs and PMIDs of all publications in a larger, field-delineated input corpus with all revisions of all articles in the English Wikipedia (Study (b)). From all potentially suited Wikipedia articles we select the most relevant ones and extend the first study by matching all publications in the large field-delineated corpus against the selected articles (Study (c)) and evaluate all heuristics. Fig. 1 gives an overview of the research design.

### Materials

The *Accounts Corpus* comprises publications referenced in predominantly secondary literature formats, such as reviews and short communications. These accounts present and discuss the development of CRISPR, providing experts’ and stakeholders’ perspectives on which publications were relevant for the innovation process. To create this corpus, we searched Google Scholar for the terms "crispr history", "crispr development", and "crispr discovery" and, based on Google's relevance ranking, reviewed the search results pages until no relevant publications were found in a series of consecutive pages. 13 publications that contain history sections were discarded because they did not focus on the history of CRISPR. The resulting sources were supplemented by three CRISPR timeline web resources, obtained by searching for "crispr timeline" on Google and reviewing the search results pages accordingly. We extracted the references from the 29 resulting sources via WoS or extracted the references manually in the case of the timeline documents, and supplemented this copus by the sources themselves.

In order to represent a topical perspective, publications containing "crispr"—as a highly distinctive term—in the title are searched in WoS and are defined as the core field. As a second layer, we add publications that contain "crispr" in their abstracts. For a third layer, representing influences and effects, we delineate publications where the proportion of references or citations to the core field compared to the total references or citations is higher than 30 percent and thus forms a substantial connection. The number of publications in this *Field Corpus* amounts to *20,585*.^4^ From this corpus, another smaller and more specific one is derived: the *WoS Highly Cited (HC) Corpus*. To provide an impact perspective, especially in contrast to the historically oriented accounts, we sort this corpus by absolute citation counts and cut off at 500 publications. This number corresponds to the volume of references in the central article (whose uncleaned number amounts to 455 titles, see below). The reason for not using citation windows here is due to the particular dynamics of our 'innovation setting', where early publications are not necessarily highly cited from the start but typically contribute a considerable number of citations over time. We also decided against standard field-normalization based on WoS subject categories because of the volume of publications in high-impact multidisciplinary journals. The corpora of *1183* Accounts and *500* HC publications overlap: The deduplicated number of publications is *1340***,** 343 publications are contained in both corpora. 309 publications of the historical Accounts Corpus are not part of the field delineation. Both corpora are exploratory, and, to some extent, pragmatically defined, as we do not yet know much about what determines reference structures in Wikipedia, especially in case of innovations.

All revisions of the CRISPR article were downloaded using the MediaWiki API, collecting the HTML versions of each revision’s page (2123 revisions as of 31 May 2021). Each revision has a unique revision timestamp, text sections (headings, paragraphs, captions, tables, lists), the reference sections (*References*, *Further Reading*), and additional metadata.

### Pilot study (Study (a))

**Fig. 2:**
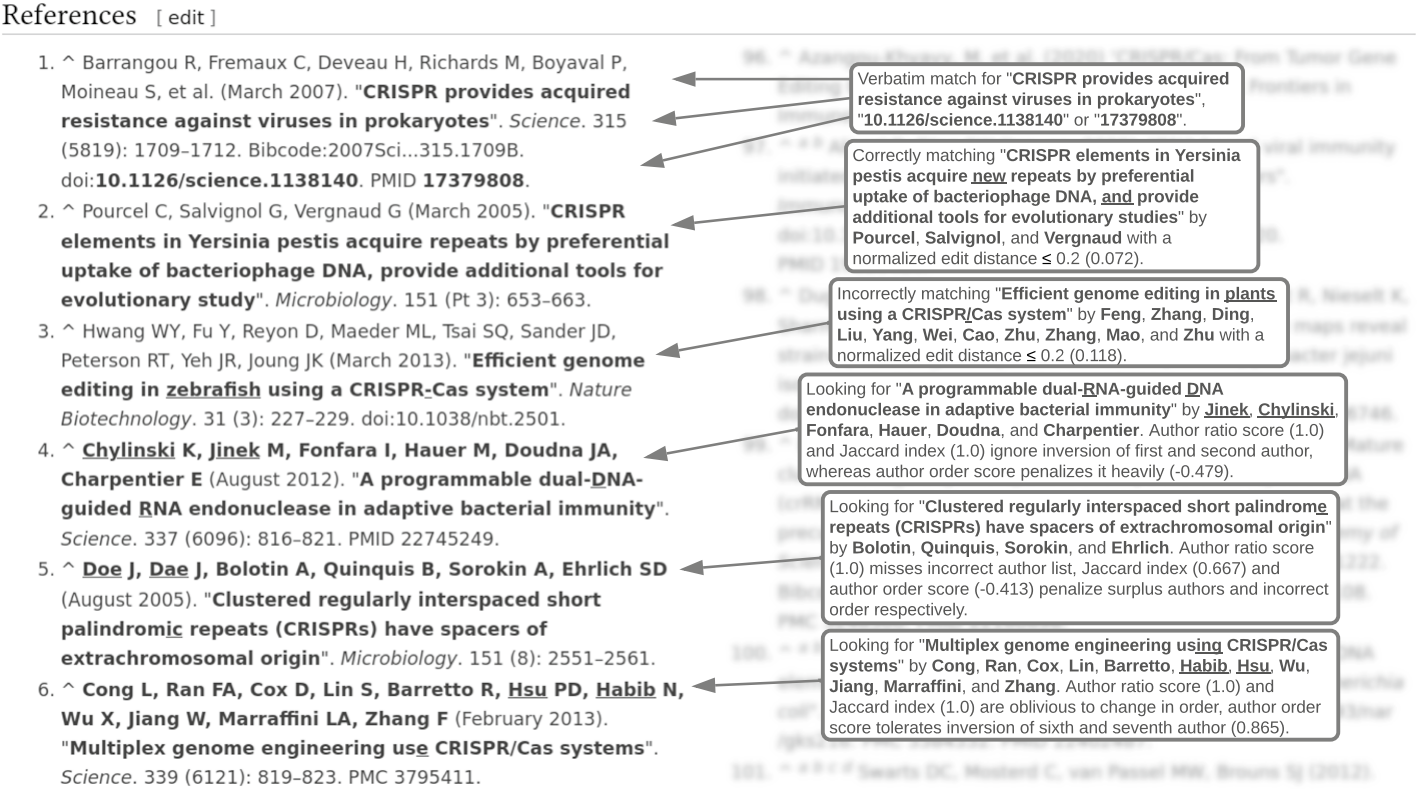
Examples showcasing the verbatim and fuzzy heuristics; divergent data underlined

In the first step, we develop and validate heuristics for matching publication corpora with the English Wikipedia in a pilot study. To cope with typos (which are often inserted by editors) we developed heuristics for literal and fuzzy reference matching with varying degrees of precision. Verbatim heuristics match titles, DOIs, and PMIDs of the publications against the entire article text including all references. All strings are converted to lowercase ASCII, alphanumeric normalization is additionally used for title matching. The fuzzy matching heuristics match the publications’ titles with extracted references and allow for normalized edit distances of 0.2, 0.3, and 0.4. In particular, they consider author matching strategies such as the publication-to-reference-author ratio, the Jaccard Index of publication and reference authors, and a new author order score. In the new score, each author of the publication is assigned a gain equal to its position in the inverted list of authors divided by its actual position, with the sum of all values being the ideal score. Then, the authors of a given reference are evaluated in turn, receiving the same value if they match the author of the publication in the respective position and losing that value if not. The author order score is normalized by the ideal score. Figures 1 and 2 shows all matching heuristics at work. Due to their idiosyncratic nature, DOI and PMID matches are accepted without further verification; verbatim title as well as all fuzzy heuristics are validated independently by the authors for all cases not identified by either DOI or PMID in the same revision.

The computation of the exact precision and recall values for the heuristics requires a manual analysis of the 2123 revisions and had to be omitted. Instead, we use an approximate value for the total number of correct items based on an analysis of references extracted from the central CRISPR article, which is primarily intended for the relative assessment of the heuristics. We also need to assess whether and to which extent we have missed referenced publications and hence analyze the references that are not in the input corpora. To this end, we manually checked and deduplicated all titles, DOIs, and PMIDs extracted from all references throughout the article’s entire revision history, resulting in 327 DOIs, 308 PMIDs, and 455 titles. We examined this data using a reverse matching to WoS and Scopus: Titles are again matched in alpha-numerical lowercase ASCII, DOIs and PMIDs verbatim, ambiguous results (mostly involving meeting abstracts and articles) are manually resolved. The respective Scopus item IDs are mapped to WoS, and the remaining title strings are manually searched on the Internet and in WoS, resulting in 333 unique WoS items, including one false positive. The comparison of matching and reverse matching does not show false negatives, and only one publication was missed by the reverse matching. Although this is almost completely consistent with our matching, it must be taken into account that the reverse matching is based on the extraction of titles, DOIs and PMIDs and is therefore not completely independent of the original matching.

### Candidate article retrieval (Study (b))

In a second step, we select, in addition to the central article, a set of ten CRISPR articles that are also highly relevant to the topic. This selection is based on knowledge about the matching heuristics plus further analytical steps.

From the pilot study we learn that the number of matched publications can hardly be improved when applying the heuristics DOI, PMID, and title in a combined fashion; in fact, DOI and PMID are already very effective. We hence perform a search of the complete Field Corpus across the entire Wikipedia revision history to find further articles relevant to CRISPR with these two identifiers. The analysis of the revision history dump (about 678 million revisions) from June 2021 for 20,585 publications represented by 18,283 DOIs and 16,973 PMIDs resulted in 849 candidate articles.

**Fig. 3:**
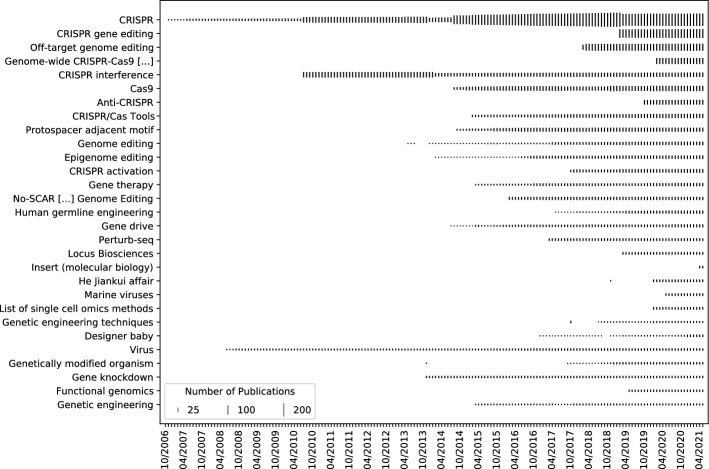
Articles in the Wikipedia dump from June 2021 for which more than five publications from the bibliometric field delineation of 20,585 publications match the DOI or PMID over their entire revision history. The articles are sorted from top to bottom by descending number of maximum matched publications. The bars indicate the maximum number of publications for each month of their revision history

From this set we discard Wikipedia articles with less than six hits and articles about persons and visualize the hit frequency and its dynamics for the remaining Wikipedia articles candidates (see Fig. 3). In an additional qualitative step, we analyze the relations between the term "CRISPR" and the main terms of the candidate articles by extracting (1) definition sentences that formulate a relationship to CRISPR from the article texts, and (2) taxonomic information from tables and tables of contents. From this information we derive a basic semantic structure consisting of applications, alternative methods of genome editing, biological principles, tools, CRISPR mechanism, and general terms, from which in turn a final set of ten additional articles is determined. We compare this set of candidate articles to publicly available datasets that are based on recent extractions of Wikipedia references. The comparison shows that these datasets are partially outdated and hence incomplete: 613 of the 849 candidate articles would be missing from the Redi and Taraborelli (2018) dataset. More importantly, out of the 11 articles in the final selection four were created after this dataset was published. 70 Wikipedia articles from the Redi and Taraborelli dataset are missing in our dataset. Spot checks show that many come from Wikipedia articles in other languages; also, several of the titles are redirects or former page names. The more recent dataset from Singh et al. (2021) still misses 368 of our candidate article set. We miss six Wikipedia articles that they would retrieve based on our Field Corpus.

### Extended matching and citation latency analysis (Study (c))

The initial matching procedures based on verbatim and fuzzy heuristics are repeated, but now using the complete Field Corpus as well as the central CRISPR article and the 10 additional articles.

## Results

### Methodical results

Table 1 shows for the central CRISPR article and the two initial, small publication corpora (the Accounts Corpus and the HC Corpus) the numbers of matched publications along with the respective precision and recall values, calculated based on the manual validation procedure described above.

**Table 1:** Evaluation of the reference matching heuristics applied to the revision history of the CRISPR article for the two corpora of relevant CRISPR-related publications

Reference matching heuristic		Accounts corpus			HC corpus		
		Matches	Precision	Recall	Matches	Precision	Recall
Exact matching heuristics							
(a) Title		172	0.994	0.934	130	1.000	0.935
(b) DOI		177	1.000	0.967	135	1.000	0.971
(c) PMID		177	1.000	0.967	135	1.000	0.971
Fuzzy matching heuristics							
(d) Title edit distance ≤ 0.2		181	0.983	0.973	137	0.985	0.971
(e) Title edit distance ≤ 0.3		185	0.951	0.962	140	0.957	0.964
(f) Title edit distance ≤ 0.4		202	0.847	0.934	152	0.849	0.928
(g) Title edit distance ≤ 0.4 + author ratio score = 1.0		163	0.976	0.869	123	0.984	0.871
(h) Title edit distance ≤ 0.4 + author Jaccard index ≥ 0.8		157	0.987	0.847	122	1.000	0.878
(i) Title edit distance ≤ 0.4 + author order score ≥ 0.8		164	0.988	0.885	125	1.000	0.899

A revision cites a publication if any of the matching heuristics correctly identifies the publication in the revision. The number of unambiguous, correctly matched publications from both corpora (Accounts and HC) is 199. The fuzzy matching heuristics (*d*), (*e*), and (*f*) identify more publications than the verbatim ones but come at the price of precision. The comparably smaller number of matches of the fuzzy heuristics (*g*), (*h*), and (*i*) result from the fact that author lists in Wikipedia articles are less well-maintained than identifiers and titles. However, the combined author and title heuristics performs better in terms of precision. The verbatim matching of publications based on titles only can lead to false positives if the title of a publication is a substring of the title of another publication (applies to one publication in the Accounts Corpus).

We now turn to the newly delineated set of ten additional CRISPR-relevant Wikipedia articles, which are analyzed for all 20,585 Field publications. Since only four publications from the Accounts Corpus, which had been initially successfully matched with the central CRISPR article, are not included in the large Field Corpus, we now work on the broader basis of the entire field instead. Altogether, 459 unique correct publications matched by the Field Corpus on all eleven articles could be identified.

Table 2 shows comparable match counts and precisions for all heuristics across the 11 articles: With the exception of "CRISPR/Cas Tools", the heuristics (*d*), (*e*), and (*f*) match more publications than the verbatim heuristics at the price of precision; heuristics (*g*), (*h*), and (*i*) cannot identify more publications than the verbatim heuristics. In general, a heuristic that relies only on the publications’ titles tends to identify more publications. This is because we match titles not only in the references but also in text segments, and treat matches of conference abstracts and corrections to articles as false positives.

**Table 2:** Evaluation of the reference matching heuristics using the Field Corpus, along with the number of matches for all eleven articles relevant to CRISPR as well as mean and median precision

Number of matched publications in each article per heuristic												Precision		
	CRISPR	CRISPR gene editing	Off-target genome editing	Genome-wide CRISPR-Cas9 knockout screens	CRISPR interference	Cas9	CRISPR/Cas Tools	Protospacer adjacent motif	CRISPR activation	Perturb.-seq	Restrict. enzyme	Mean	Median	StD
(*a*)	292	110	52	43	57	50	21	18	16	9	6	0.929	0.944	0.044
(*b*)	279	102	54	41	50	45	21	18	15	8	4	1.000	1.000	0.000
(*c*)	271	103	50	41	57	44	21	18	15	8	4	1.000	1.000	0.000
(*d*)	311	114	65	46	63	52	20	20	16	9	7	0.865	0.889	0.107
(*e*)	346	127	76	52	72	57	21	24	16	11	9	0.764	0.790	0.126
(*f*)	483	218	112	82	98	109	23	39	18	21	14	0.526	0.462	0.174
(*g*)	275	104	60	43	48	47	21	19	14	3	7	0.881	0.907	0.107
(*h*)	255	93	52	41	34	40	18	19	12	0	4	0.948	0.950	0.033
(*i*)	267	104	53	43	39	42	20	19	14	1	4	0.943	0.950	0.042

Summarizing the results at the level of individual matches, i.e., counting a publication that was correctly found in more than one of the 11 articles multiple times, we could identify 516 out of 670 matches by their exact title, 52 by their DOI, 91 by their PMID, 10 by heuristic (*d*), and one by heuristic (*f*). Counting at the level of distinct publications and pooling the results, 459 unique publications out of 20,585 in our corpus were correctly matched from all matching heuristics, with 458 publications found using the exact methods and 451 found using the two identifiers PMID and DOI only. By matching with title only we found 432 publications, with DOI only 444 publications, and with PMID alone 429 publications.

These results confirm the finding from the pilot study, namely that the gain from more elaborate procedures in terms of matched publications is marginal, and that matching with PMID and DOI only is a good compromise between accuracy and simplicity. Additional matches resulting from the exact title heuristics are bought with a reduced overall precision.

To assess potential benefits of the relaxed matching heuristics in terms of delays, we also calculated the mean delay in days between each pair. Table 3 shows for each heuristic (rows) the average delay in days between the time it matches a publication and the time the same publication is matched by another heuristic (columns). Detailed calculations (see Table 5 in the Appendix) show that all fuzzy title-only matching heuristics ((*d*), (*e*), and (*f*)) are able to identify publications earlier than the PMID-based approach in 25%, and earlier than the DOI-based approach in about 12% of all cases where the respective heuristics correctly match a publication. When looking at the mean delays (positive = slower, negative = faster) in Table 3, the fuzzy heuristics are on average only nine days faster (PMID, italic) or even about one day slower (DOI, bold) than the respective verbatim identifiers.

**Table 3:** Mean delay in days between heuristics, calculated over all eleven articles

Comparison of Reference Matching Heuristics									
	(*a*)	(*b*)	(*c*)	(*d*)	(*e*)	(*f*)	(*g*)	(*h*)	(*i*)
(*a*)		8.3	− 1.9	5.4	5.4	5.3	− 19.3	− 54.5	− 39.3
(*b*)	− 8.3		− 8.4	**− 1.2**	**− 1.2**	**− 1.3**	− 28.9	− 65.8	− 49.0
(*c*)	1.9	8.4		*9.2*	*9.2*	*8.9*	− 26.6	− 58.0	− 36.6
(*d*)	− 5.4	**1.2**	* − 9.2*		0.0	0.0	− 25.2	− 62.4	− 45.0
(*e*)	− 5.4	**1.2**	*− 9.2*	0.0		0.0	− 25.2	− 62.4	− 45.1
(*f*)	− 5.3	**1.3**	* − 8.9*	0.0	0.0		− 25.2	− 61.3	− 45.2
(*g*)	19.3	28.9	26.6	25.2	25.2	25.2		− 31.5	− 19.3
(*h*)	54.5	65.8	58.0	62.4	62.4	61.3	31.5		14.0
(*i*)	39.3	49.0	36.6	45.0	45.1	45.2	19.3	− 14.0	

There is considerable variance between articles. For example, the fuzzy title heuristics ((*d*)–(*f*)) are able to find publications in "Genome-wide CRISPR-Cas9 knockout screens" earlier than the DOI- and PMID-based heuristics in about 90 percent of all cases. For all other articles, the fuzzy title heuristic is better than the DOI heuristic in less than 10% of the cases. For eight articles, fuzzy title heuristics succeed in finding a publication earlier than PMID in 25% or less of the time, and for two articles in about one third of the time. If wording heuristics are used exclusively, almost all publications are found no later than three days later.

Some articles such as "CRISPR/Cas Tools" and "Restriction enzyme" also show considerable variance when compared to other heuristics, but, the advantages of the fuzzy heuristics over individual verbatim heuristics disappear when verbatim heuristics and relaxed heuristics are compared at the aggregate level, as can be seen in Table 4: With the exception of "Restriction enzyme", "Genome-wide CRISPR-Cas9 knockout screens" and, to a lesser extent, "CRISPR", relaxed heuristics find publications later than verbatim heuristics.

**Table 4:** Comparison of delays at verbatim-relaxed level

	CRISPR	CRISPR gene editing	Off-target genome editing	Genome-wide CRISPR-Cas9 knockout screens	CRISPR interference	Cas9	CRISPR/Cas Tools	Protospacer adjacent motif	CRISPR activation	Perturb.-seq	Restriction enzyme
Verbatim matching earlier than relaxed in %	25.2	2.0	0.0	2.4	24.1	55.6	0.0	0.0	21.4	37.5	25.0
Relaxed matching earlier than verbatim in %	0.4	0.0	0.0	19.0	0.0	0.0	0.0	0.0	0.0	0.0	25.0
Mean delay between earliest verbatim and earliest relaxed heuristic in days (StD.)	− 48.7	0.0	0.0	− 0.5	− 117.8	− 116.3	0.0	0.0	− 35.7	− 0.1	− 76.7
	(139.7)	(0.0)	(0.0)	(1.4)	(223.6)	(160.9)	(0.0)	(0.0)	(128.3)	(0.1)	(195.9)

The variance between the selected Wikipedia articles in the results of the matching heuristics suggests that the editing of similar articles is not structurally coordinated. However, missing metadata seems to be quickly added by the community, with the combination of verbatim identifiers sufficiently covering minor inconsistencies and errors in the referencing strategies of individual editors. This finding complements the (rather anecdotal) evidence presented by Benjakob and Aviram (2018) regarding editors’ constant reviewing processes. Altogether, the delays of heuristics relative to each other are quite small and thus the temporal perspective does not change our earlier conclusion that a combination of all three exact heuristics is optimal, while matching with DOI and PMID only offers a good compromise between recall and ease of implementation.

Caution is advised when titles are matched against the entire text of an article (as was done here): Some publication titles are exact substrings of other publications, others are so short that they can literally appear in the article. On the other hand, individual publications are referred to in the text of the Wikipedia article rather than in the reference section, which can be found only by a full-text search for the titles.

With the extensive validations and mutual comparisons of the heuristics, we have achieved a high confidence in the correctness of matching publications as well as of the date of their first occurrence. In the following assessment of the central CRISPR article and the related Wikipedia articles in terms of the structure of references and the timeliness of citations, we refer to the first occurrence as the earliest date on which a reference was correctly identified, regardless of the applied heuristic.

### Empirical results

We first focus on the reference structure of the central CRISPR article. By reverse matching all extracted references from this article, we are able to correctly identify 332 WoS publications across all revisions. 199 of those match one or both of the initial corpora, the Accounts Corpus, and the HC Corpus: 183 with the Accounts Corpus versus 139 with the HC Corpus. In total, 294 of the 332 referenced WoS publications (excluding one that was missing from the reverse procedure) are part of our bibliometric field delineation of 20,585 publications. 99 of these are neither part of the HC nor the Accounts Corpus. Most of these publications belong to the core of the Field Corpus (the term CRISPR appears in the title) and belong to the time segment from 2015 onwards.^5^ At the same time, this more recent time segment is also less covered by our historical accounts, whose publication years are typically between 2015 and 2018. Very specific and little-cited publications are also featured. The phenomenon that such less eminent publications are cited in the Wikipedia article may be explained by the fact that citations on Wikipedia and citations within the scholarly publication system fulfill different functions: The latter are an indicator of the extent to which claims stimulate further research, whereas in Wikipedia the function of citations is exclusively to substantiate knowledge claims: A hardly cited publication that supports a very specific claim may be appropriate from the functional logic of the Wikipedia article.

38 referenced publications are WoS source items but not part of the Field Corpus, although four of this subset belong to the Accounts Corpus. 14 are at least loosely connected to the Field Corpus.

Of the 332 WoS publications, 224 have the WoS document type 'Article' and 64 'Review', others are Editorials, News Items, and Letters.

Of the remaining non-WoS references, about 110 could be identified through manual search as contributions to popular science or technical journals (such as "Technology Review"), technical blogs, and news sites. There are also references to clinical trials and patents as well as to other web resources. Although we have not deepened our analysis here, based on manual inspection it is likely that these references also support CRISPR-specific content claims in a narrower sense and not other areas (such as legal or ethical aspects). This does also fit our observation that (in single cases) references to scientific publications do not follow the standard format but refer to recommendation sites,^6^ both of which are indications for not strictly following a scientific referencing policy.

It can be concluded that, on the one hand, the Wikipedia article reflects a broader discourse than a regular scientific review since it covers popular science and journalistic media, even if these sources are still relatively marginal. Our observation confirms the tendency of the findings of Benjakob and Aviram, who also report a minority of other, non-published sources, in their case books and websites.

On the other hand, of the 60 publications referenced in more than five of our 32 historical accounts, 48 are referenced in both the central and related Wikipedia articles. Similarly, in the HC Corpus, 46 of the 60 most cited publications are referenced in at least one of the selected Wikipedia articles. This suggests that the opinion of Wikipedia editors on the relevance of publications is broadly in line with the scientific community and the metric perspective. The number of references of the central Wikipedia article, for example, is in the same order of magnitude as that of a scientific review. Nevertheless, the selected historical accounts also differ from each other to a certain extent as they are from different years and slightly different emphases are likely. Against this background, the fact that 183 of 332 WoS publications (55%) referenced by the central CRISPR article match the Accounts Corpus is a convincing sign that Wikipedia editors largely adhere to strictly scientific formats.

**Fig. 4:**
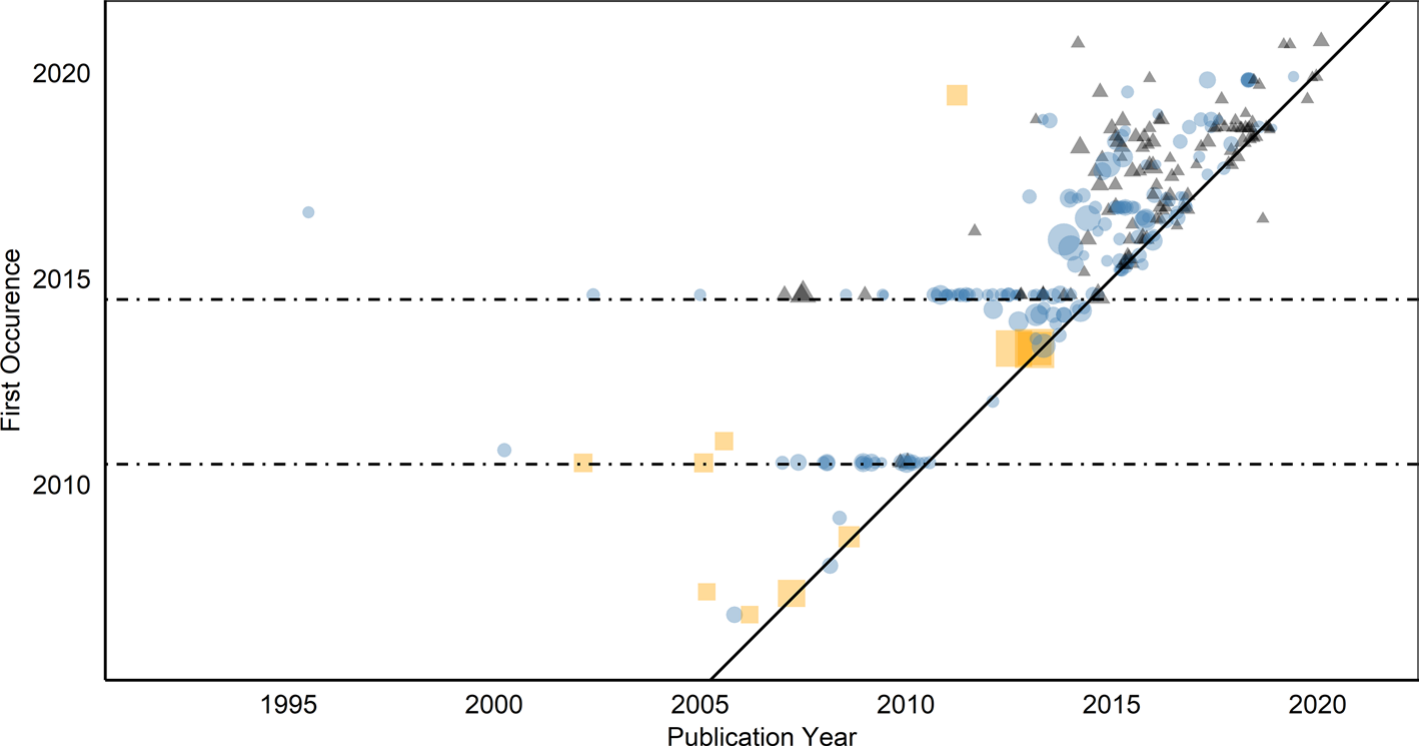
Publication dates (x-axis) of matched publications in relation to the first occurrence on Wikipedia (y-axis) of the central CRISPR article

Figure 4 shows all references based on the large field corpus matched to the central CRISPR article: It plots the relation between publication years (as indexed in WoS) and the first occurrence in the central CRISPR article. The WoS publication dates used here are based on the volume/issue data; online advance data were added to WoS relatively recently and are not available in WoS for our dataset. The point size represents the number of citations in WoS, orange-colored squares represent a couple of seminal works that have particularly shaped the innovation dynamic,^7^ blue points represent publications from the historical accounts corpus, and black triangles all others. Transparency is used to show points lying on top of each other. Publications from the historical accounts dominate the older time segment. It is noteworthy that the article exists for a period of one and a half years without any reference: The first version of the CRISPR article dates from 30 June 2005, whereas the first referenced publications occur in November 2006. There are substantial delays of up to several years in earlier revisions. Two distinct temporal stages can be observed, 2010 and 2014 (indicated in the graph by dashed lines), in which a number of older publications are retroactively referenced. First, the year 2010 coincides with the introduction of a 'History' section in the central CRISPR article. Second, the key publications of the innovation date from 2011 to 2013, but we see some older publications appearing for the first time in 2014. However, some highly-cited and seminal publications from the periods before and between these years were referenced more quickly: In 2006, 2007, and 2008, fundamental works on CRISPR as a defense system in prokaryotes are referenced within the same year. Central publications by group leaders Doudna and Charpentier (Jinek et al., 2012) as well as Zhang (Cong et al., 2013) and Church (Mali et al., 2013), introducing the applicability for genome editing, occur in the Wikipedia article on the same date in April 2013. However, the delayed reference of further publications suggests that the importance of the development was recognized somewhat late. In any case, the article’s presentation of the research strand is revised and expanded from 2014, leading to a reappraisal of older work. In comparison, Benjakob and Aviram (2018) found (on a more qualitative basis) that the full integration of key publications may depend on how long it takes for the field to reformulate its central paradigm and to generalize the findings.

**Fig. 5:**
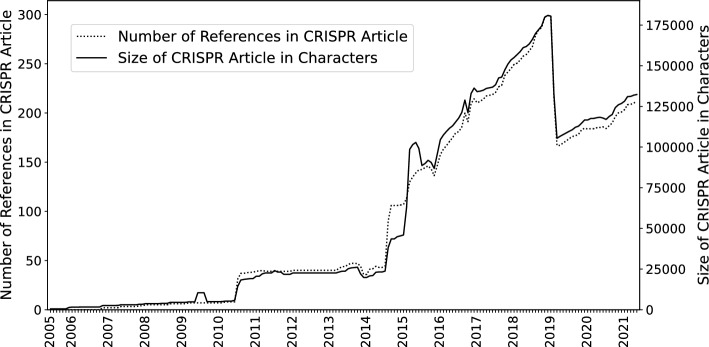
Smoothed dynamics of the growth of text of the CRISPR Wikipedia article and its references

These temporal dynamics also emerge from a broader quantitative perspective, which results from the CRISPR article’s character and reference numbers:

Figure 5 shows the dynamics of the central CRISPR article in terms of text size in characters as well as number of references. The strong parallelism of text and reference graphs suggests that the new claims are indeed supported by newly introduced references, as required.

Both graphs show the first substantial step in 2010 and another larger increase from 2014 onwards, demonstrating that text and references are added in a substantial manner. In 2019, the application-related section is moved to a new article on CRISPR gene editing, leading to a decrease of text and references. Thus, the late recognition of publications may to some extent be consistent with the general dynamics of the article’s edition.

**Fig. 6:**
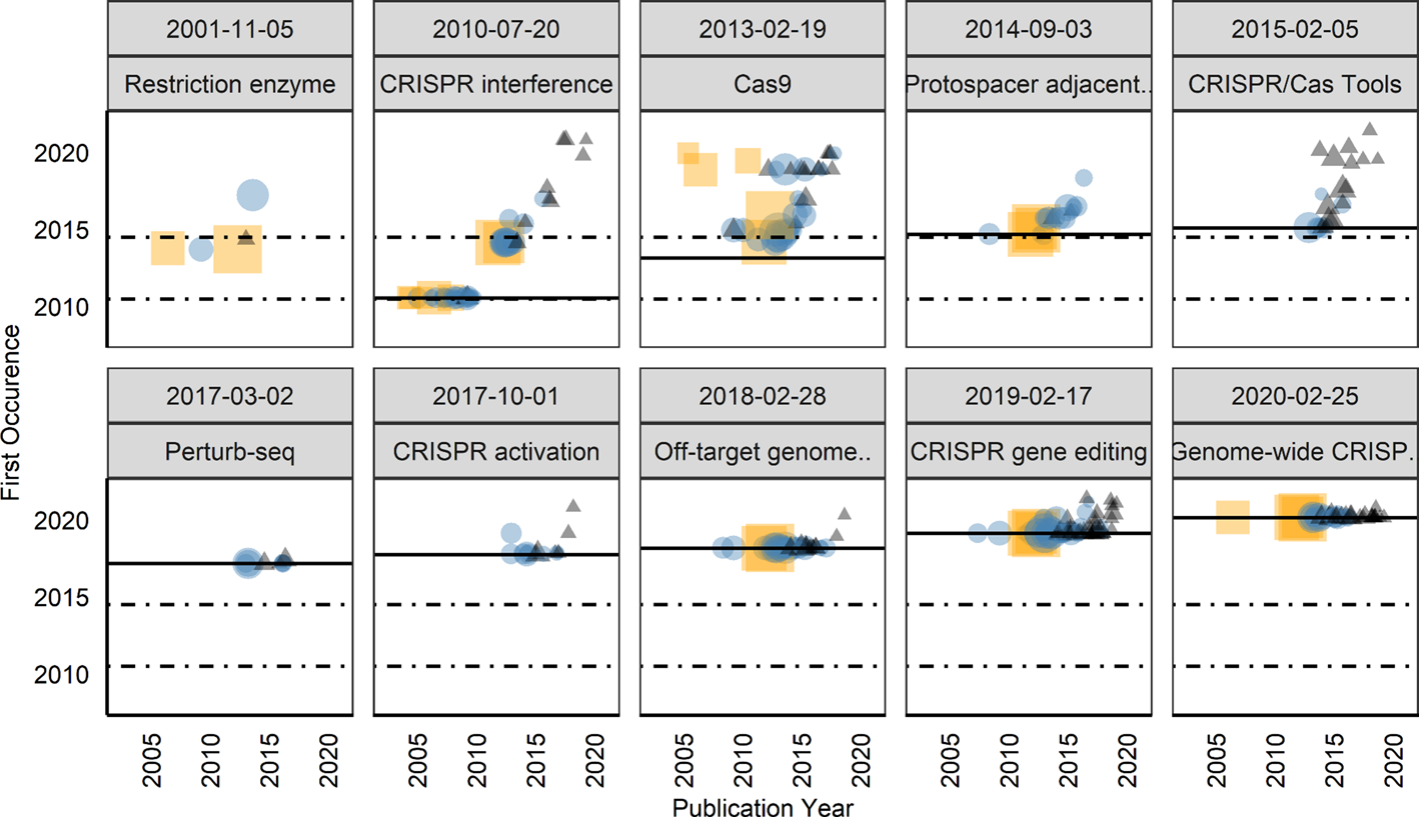
Publication dates (x-axis) of matched publications in relation to the first occurrence (y-axis) of the selection of CRISPR-related articles

Figure 6 complements the perspective of the central CRISPR article with that of the ten selected related articles, sorted by the creation dates of the articles. Again, the point size represents the citation count in WoS, and the two dashed horizontal lines represent the two main peaks of the CRISPR article dynamics, while the solid horizontal line represents the article creation dates (omitted for "Restriction Enzyme"). Orange squares represent a couple of seminal works, blue points represent publications from the historical accounts corpus, and black triangles all others.

We observe that four CRISPR-related articles were newly created between 2010 and 2014 (i.e., before or at the time of the accelerated growth of the central CRISPR article from 2014): (1) "CRISPR interference" was created in 2010 and is the first that describes the biological mechanism in bacteria, while the technique was introduced in 2013. (2,3) "CRISPR/Cas Tools" and "Cas9" are both from February 2013, with the former being the first application-oriented article. Cas9 refers to a protein that is relevant for the immunological defense of bacteria and is crucial for the CRISPR/Cas gene-editing technique. (4) "Protospacer adjacent motif", which is based on the biological principles of the CRISPR/Cas mechanism, was created in September 2014. The oldest article, "Restriction Enzyme", about the enzyme essential for the bacterial immune system underlying the technique, is from 2001.

The five newer articles are more application-oriented. While the five older articles show different patterns of delayed and instant recognition, the dynamics of referencing in case of the five newer articles in the second row are largely dominated by back-referencing to publications published before the time the Wikipedia articles were written. Thus, on the one hand, the number of specialized Wikipedia articles grows as the innovation and its application potential unfolds. On the other hand, new idiosyncratic dynamics are emerging to a certain extent: In the case of the article "Cas9", we see that a number of publications is retro-indexed in 2019.

Of the total 459 distinct correctly matched WoS publications based on the 11 articles, 162 occur in more than one Wikipedia article. Of these 162, 49 distinct publications (30%) occur with at least one delay in the publication’s first occurrence between Wikipedia articles, not including delays caused solely by a later creation date of the respective article. In the majority of cases, publications occur either on the same day as the first occurrence (within our set) or on the creation date of the respective article.

**Fig. 7:**
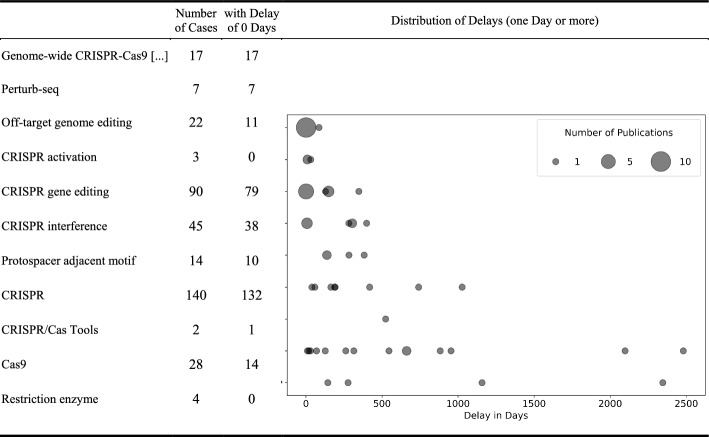
Delays (in days) of occurrences in Wikipedia articles in relation to other articles within the set

Table 5/Figure 7 gives a more detailed insight into delays between the selected Wikipedia articles. The delays were normalized with the creation date of the respective articles. If a paper is not added to Wikipedia on the same day it is published (and neither on the Wikipedia article’s creation date), it is often added within a few days later, indicating that newly recorded references have been copied from one article to another. In some cases, however, references are entered on some Wikipedia articles months or even years after their first occurrence on another article. The central CRISPR article also has some delays in relation to related Wikipedia articles; longer delays relate to three publications that occur on the CRISPR site in 2017 and 2018 but were already referenced in 2014 and 2015 in the articles Cas9 and CRISPR interference. It can be concluded that reference entries in related Wikipedia articles are usually in close temporal proximity to each other, but, apparently, they are not structurally coordinated.

**Fig. 8:**
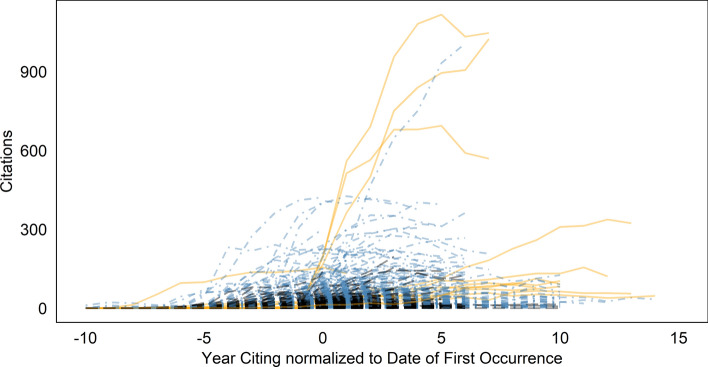
Matched publications’ yearly citation counts are plotted in relation to the date of first occurrence in the CRISPR article as baseline

How does the speed with which Wikipedia recognizes publications relate to the speed in which the scientific community picks up publications? Figure 8 shows the comparison between the first occurrence in Wikipedia (based on all 11 articles) and the citation curves of the publications, normalized to the date of the first occurrence of the publications, so that the different publication dates are hidden. Citations before this date have negative numbers. Orange solid lines represent seminal works, blue dashed lines represent publications from the Accounts Corpus, and black long-dashed lines all others.Noticeably, Wikipedia is quite fast with regard to almost all seminal publications: They first occur clearly before the peak of the citation distributions, in some cases especially early in relation to very long citation curves, indicating late, but long-term recognition by peers. Wikipedia editors obviously recognize and cite publications broadly at the same time as the scientific community.

## Discussion and conclusion

We have analyzed the use of scientific literature in the Wikipedia article on CRISPR and the timeliness of the reference patterns of this central article as well as related articles. Our goal is to assess Wikipedia’s relevance and adequacy as a medium for the representation of scientific contents and, in particular, the tracing of scientific innovations. We tested different matching procedures to map from publication corpora to all revisions of Wikipedia’s central article and related articles on CRISPR. Methodically we demonstrated that a combination of verbatim matching heuristics yields sufficient accuracy to make Wikipedia a valuable object for analyses of science communication in addition to standard bibliometric sources. With that said, there is some variance between the analyzed Wikipedia articles.

Our results are also promising on an empirical level: There is evidence that much of the CRISPR/Cas9 literature referenced in Wikipedia is highly cited or has been acknowledged by experts in the field. The majority of referenced publications is covered by WoS and part of our WoS field delineation; however, the concordance is not complete. News, magazine pieces, and other web resources are also referenced to some extent, which is contrary to the practices in regular scientific secondary or tertiary sources, at least in STEM subjects.

We observe that the timeliness of referencing improves over time in the sense that major gaps in the recognition of older literature are filled quite early (in 2010 and 2014), while delays of one to a few years also occur later on. In the innovation’s earlier phase, timeliness depends to a certain degree on the general level of Wikipedia’s editors’ activity regarding the article and the topic. We conclude that delays in Wikipedia should be viewed less as a fixed quantity and more as dependent on the respective circumstances. However, unlike databases such as WoS, whose coverage policies are based on journals rather than individual publications, Wikipedia’s reference patterns represent a degree of article-level expert recognition. In the course of innovation, the representation of the topic in Wikipedia grows both in terms of the amount of text and the number of references of the central CRISPR article, but also in terms of the number of related, more specialized Wikipedia articles. It should be considered that the 29 historical accounts—apart from two exceptions^8^—date back to the period from 2014 to 2018 or later, i.e., at the same time or later than some publications occur in the central CRISPR article. Although articles do not always occur in Wikipedia in the year of their print publication, we also find that the time of occurrence in Wikipedia precedes the citation peak to a larger extent. While the picture is not clearcut, Wikipedia performs comparatively well in terms of timeliness. Wikipedia’s policy of constant updating is of course a clear advantage compared to the fixed state of classical reviews. All mentioned factors support our initial hypothesis that Wikipedia can be considered and used as a kind of Living Review due to its extensive adherence to scientific formats and referencing standards, its timeliness, as well as its constant updates. However, largely decentralized, crowd-sourced editing procedures cause inconsistencies to a smaller extent, which can be observed in a comparative analysis of related Wikipedia articles. The articles show slight differences in the early adoption of identifiers, and publications are not always included in similar articles at the same or earliest time, suggesting that editing of similar articles is not necessarily coordinated.

Benjakob and Aviram (2018) relied on a manual text-analytical approach in search for editorial trends and observed no relationship between number of edits and text size. By contrast, the latter metric (as well as number of references) proved in our case to be an explanatory resource for certain patterns of delays. Metrics can therefore be an insightful resource for understanding specific dynamics.

We are working with print publication dates. While in our case spot-checks found very few preprint-versions of publications referenced in the central Wikipedia article, this may be different in other fields and with more recent data. Comparing Wikipedia occurrence data with these and online advance dates can make the analysis of latency more precise and nuanced.

Another limitation of our research is that, as a case study, it focuses on the specific context of a novel-prize winning innovation from a STEM subject. It is not unlikely that the high profile of the innovation attracts a considerable number of editors, which could affect the dynamics of editing and latency of references. Similarly, the fact that the innovation and the related debates were so widely discussed in non-scientific sources such as newspapers and technology magazines could have impacted the extent to which these sources also occur as references. Given this limitation of a high-profile case and our finding that citation latency in this case is influenced by the specific interaction of a subfield's development and editor activity, future research could investigate whether (a) there is high profile research with low coverage in Wikipedia, (b) there is low profile research with high coverage in Wikipedia, and (c) there are different trends in STEM, humanities, etc.

Within the framework of our explorative, descriptive design, we also did not specifically investigate the question of whether the representation of the field in Wikipedia in this case leads to feedback effects on intra-scientific communication, i.e., whether the early occurrence of some publications in Wikipedia leads to more attention and more citations within the scientific community. One may interpret Fig. 8 to mean that this is possible to a certain extent (though not necessarily true), because such effects presuppose that occurrences in Wikipedia are early, and many are certainly before the citation peak. Proving a causal effect would be the task of further research, probably based on the analysis of text similarities, i.e., specific word usage patterns (Thompson & Hanley, 2018).

## Figures and Tables

**Table 5:**
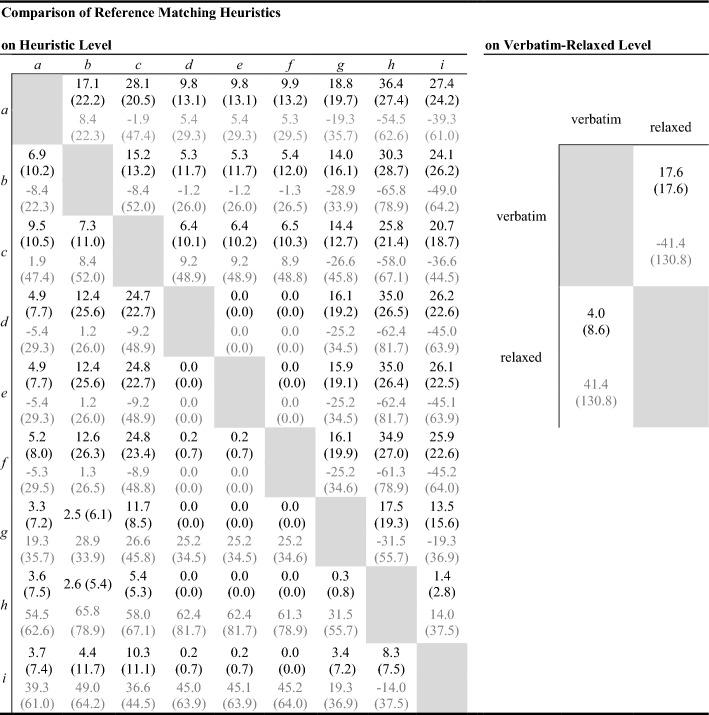
Rate at which heuristic in row identifies heuristic in column earlier in black, mean delay in days between heuristics in gray, standard deviation for both in parentheses

## Appendix

See Table 5.

## Footnotes

1 https://en.wikipedia.org/wiki/Wikipedia:Reliable_sources#Primary,_secondary,_and_tertiary_sources.

2 Our paper is part of a larger project on science-communication through Wikipedia, with another project on CRISPR-related controversies about micro-notability on Wikipedia.

3 https://en.wikipedia.org/wiki/CRISPR.

4 On a frozen version of the WoS raw database from April 2020, to ensure reproducibility. Subsequent calculations, e.g., citation counts, are based on the respective version from April 2021.

5 The decision against citation windows and in favor of cumulative citation counts, by which we favored early publications with long-term influence, could turn into the opposite here.

6 https://facultyopinions.com/prime/718276719#eval793490932.

7 Selected on the basis on multiple occurrences in secondary accounts, as well as information in the current version of the history section.

8 These are publications from 2012 and 2013, originating from within the closer group of early eminent CRISPR researchers such as Koomin and Barrangou.
